# Complex genotype–phenotype correlation of *MYH11*: new insights from monozygotic twins with highly variable expressivity and outcomes

**DOI:** 10.1186/s12920-024-01908-5

**Published:** 2024-05-21

**Authors:** Xiaojiao Wei, Yunting Ma, Bobo Xie, Chunrong Gui, Meizhen Shi, Xianda Wei, Yan Huang, Xin Fan, Qiaozhen Wei, Qingmei Huang, Li Deng, Chi Zhang, Xiaoli Deng, Baoheng Gui, Yujun Chen

**Affiliations:** 1 The Second School of Medicine, Guangxi Medical University, Nanning, China; 2 Center for Medical Genetics and Genomics, The Second Affiliated Hospital of Guangxi Medical University, Nanning, China; 3 The Guangxi Health Commission Key Laboratory of Medical Genetics and Genomics, The Second Affiliated Hospital of Guangxi Medical University, Nanning, China; 4 Department of Pediatrics, The Second Affiliated Hospital of Guangxi Medical University, Nanning, China; 5 Department of Obstetrics, The Second Affiliated Hospital of Guangxi Medical University, Nanning, China; 6 Department of Ultrasonic, The Second Affiliated Hospital of Guangxi Medical University, Nanning, China

**Keywords:** Familial thoracic aortic aneurysm, Patent ductus arteriosus, *MYH11*, Genotype–phenotype correlation, Monozygotic twins, Expressivity

## Abstract

**Background:**

Thoracic aortic aneurysm/dissection (TAAD) and patent ductus arteriosus (PDA) are serious autosomal-dominant diseases affecting the cardiovascular system. They are mainly caused by variants in the *MYH11* gene, which encodes the heavy chain of myosin 11. The aim of this study was to evaluate the genotype–phenotype correlation of *MYH11* from a distinctive perspective based on a pair of monozygotic twins.

**Methods:**

The detailed phenotypic characteristics of the monozygotic twins from the early fetal stage to the infancy stage were traced and compared with each other and with those of previously documented cases. Whole-exome and Sanger sequencing techniques were used to identify and validate the candidate variants, facilitating the analysis of the genotype–phenotype correlation of *MYH11*.

**Results:**

The monozygotic twins were premature and presented with PDA, pulmonary hypoplasia, and pulmonary hypertension. The proband developed heart and brain abnormalities during the fetal stage and died at 18 days after birth, whereas his sibling was discharged after being cured and developed normally post follow-up. A novel variant c.766 A > G p. (Ile256Val) in *MYH11* (NM_002474.2) was identified in the monozygotic twins and classified as a likely pathogenic variant according to the American College of Medical Genetics/Association for Molecular Pathology guidelines. Reviewing the reported cases (*n* = 102) showed that the penetrance of *MYH11* was 82.35%, and the most common feature was TAAD (41.18%), followed by PDA (22.55%), compound TAAD and PDA (9.80%), and other vascular abnormalities (8.82%). The constituent ratios of null variants among the cases with TAAD (8.60%), PDA (43.8%), or compound TAAD and PDA (28.6%) were significantly different (*P* = 0.01). Further pairwise comparison of the ratios among these groups showed that there were significant differences between the TAAD and PDA groups (*P* = 0.006).

**Conclusion:**

This study expands the mutational spectrum of *MYH11* and provides new insights into the genotype–phenotype correlation of *MYH11* based on the monozygotic twins with variable clinical features and outcomes, indicating that cryptic modifiers and complex mechanisms beside the genetic variants may be involved in the condition.

**Supplementary Information:**

The online version contains supplementary material available at 10.1186/s12920-024-01908-5.

## Introduction

Thoracic aortic aneurysm/dissection (TAAD) is a disease with an extremely high mortality rate [1] and is divided into three types: sporadic, syndromic, and hereditary non-syndromic [2]. The major risk factor for sporadic TAAD is hypertension, which typically occur in older adults [3]. Syndromic TAAD is often observed in syndromes of connective tissue diseases, such as vascular Ehlers–Danlos syndrome and aneurysms osteoarthritis syndrome [2, 4, 5]. The main molecular and genetic mechanisms of hereditary non-syndromic TAAD are unclear. Hereditary non-syndromic TAAD is partly caused by variants in genes that encode the contractile proteins of vascular smooth muscle cells (SMCs) [6]. Various genes, including *ACTA2*, *MYH11*, *MYLK*, and *PRKG1*, have been identified to be responsible for hereditary non-syndromic TAAD, which is typically accompanied by patent ductus arteriosus (TAAD/PDA) [6]. Hereditary TAAD/PDA is genetically heterogeneous and inherited in an autosomal-dominant manner [7].

*MYH11* (OMIM:160,745) is on chromosome 16p13.11 and encodes the heavy chain of myosin 11 protein [8]. This contractile protein is found in arterial vascular SMCs and plays a critical role in maintaining vascular wall stability [9]. variants in *MYH11* result in incorrect assembly of the myosin filament and microfilament, thereby affecting the synthetic and contractile functions of SMCs [10, 11]. Pathogenic variants in *MYH11* may result in hereditary TAAD/PDA, visceral myopathy 2, or megacystis–microcolon–intestinal hypoperistalsis syndrome [12]. The proliferation and migration of SMCs leads to the formation of intimal cushions in the ductus arteriosus after birth, and constriction of SMCs results in ductal contraction and permanent ductus closure [13, 14]. variants of *MYH11* that disrupt the formation of smooth muscle myosin heavy chains and SMC contractile function lead to hereditary TAAD/PDA [15].

Hereditary TAAD/PDA may result from pathogenic variants of *MYH11.* However, the mechanism of *MYH11* is quite complex and has not yet been completely understood. The genotype–phenotype correlation of *MYH11* has not been definitively determined. Herein, we performed phenotypic and genetic analysis of monozygotic twins in detail and further analyzed the complex genotype–phenotype association of *MYH11*.

## Materials and methods

### Whole-exome sequencing and analysis

Blood samples were collected from the proband and his parents into tubes containing EDTA for whole-exome sequencing, and genomic DNA was extracted from the blood according to the standard protocols of the DNeasy Blood & Tissue kit (Qiagen GmbH, Hilden, Germany). Human exome sequencing libraries were constructed using IDT xGen Exome Hyb Panel v2 (Integrated DNA Technologies, Coralville, IA, USA), and the generated amplicons were sequenced on an Illumina NovaSeq platform (San Diego, CA, USA) following the manufacturers’ instructions. The whole-exome sequences were analyzed and annotated using the Genome Analysis Toolkit. The sequencing fragments were compared with the reference genome of University of California Santa Cruz Genome Browser on Human (GRCh37/hg19) by Genome Analysis Toolkit HaplotypeCaller. The variation with frequency ≥ 3% in Genome Aggregation Database (gnomAD), Exome Sequencing Projec (ESP), 1000 Genomes Projec (1000G) databases and in-house database and the nonfunctional variation (such as synonymous variation and variation in non-coding region) were removed. According to the American College of Medical Genetics/Association for Molecular Pathology (ACMG/AMP) guidelines [16], the inclusion criteria for candidate variants were: [1] the phenotype of the patient was likely associated with the dominant Mendelian disease resulting from a heterozygous variant in an established causative gene; [2] the frequency of variants in the East Asian population in the databases of 1000 G, gnomAD, ESP, and our in-house database were below 0.5%; and [3] multiple *in silico* tools, such as CADD, Rare Exome Variant Ensemble Learner, Sorting Intolerant from Tolerant, and Polymorphism Phenotyping v2, indicated the variant to be deleterious.

### Sanger sequencing and analysis

Sanger sequencing was conducted to validate the candidate variants. We designed a pair of primers (5′-ACTTGACCTGTGGGGTTCTG-3′ (forward) and 5′-TAACCACTGCACCACAATGC-3′ (reverse)) to amplify the corresponding DNA fragment with the identified *MYH11* variant, and the generated amplicons were sequenced on an ABI 3500xLDX platform (Applied Biosystems, Foster City, CA, USA). The sequencing chromatograms analyzed using Chromas confirmed the presence of the variant.

### Analysis of the genotype–phenotype correlation of *MYH11*

Data on the genotypes and phenotypes of *MYH11* were obtained from databases including Clinvar (https://www.ncbi.nlm.nih.gov/clinvar/), Human Gene Mutation Database (https://www.hgmd.cf.ac.uk/ac/index.php), and PubMed (https://pubmed.ncbi.nlm.nih.gov/). The inclusion criteria of the variants were as follows: pathogenic variants of *MYH11* that were associated with TAAD/PDA or wherein the result of functional studies supported classification as a deleterious variant. Phenotypic data were derived from the pedigrees of the reported cases. The Fisher’s exact test was applied to evaluate differences in constituent ratios of specific type of variation among cases with different phenotypes using IBM SPSS Statistics 26. Statistical significance was defined as *P* < 0.05. The Bonferroni correction with an adjusted *P* value (*P* < 0.0167) was further applied to the pairwise comparison.

## Results

### Variable clinical manifestations and outcomes of the monozygotic twins

**Fig. 1:**
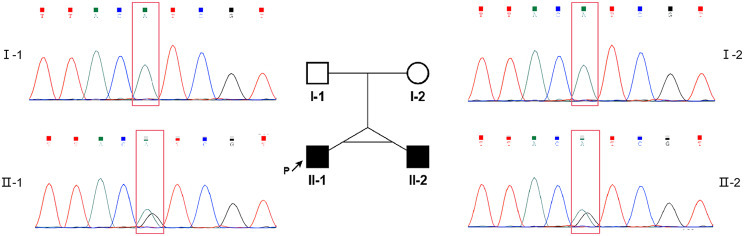
Pedigree chart and results of Sanger sequencing of the family including non-consanguineous parents and monozygotic twins. A *de novo* heterozygous missense variant, NM_002474.2: c.766 A > G p. (Ile256Val) in *MYH11* was identified in II-1 and II-2

The proband (Fig. 1, II-1) and his brother (Fig. 1, II-2) were dichorionic-sac and diamniotic-sac twins and were born by spontaneous delivery at 30^+ 3^ weeks from non-consanguineous and healthy parents (Fig. 1). Scanning and analyzing multiple genetic markers (short tandem repeat) suggested that they were monozygotic twins (data were shown in Supplementary Material S1). Their karyotypes were 46, X, inv(Y) (p11q11), which can be mentioned as polymorphism. And no abnormality was found in the noninvasive prenatal testing or prenatal copy number variation sequencing.

**Fig. 2:**
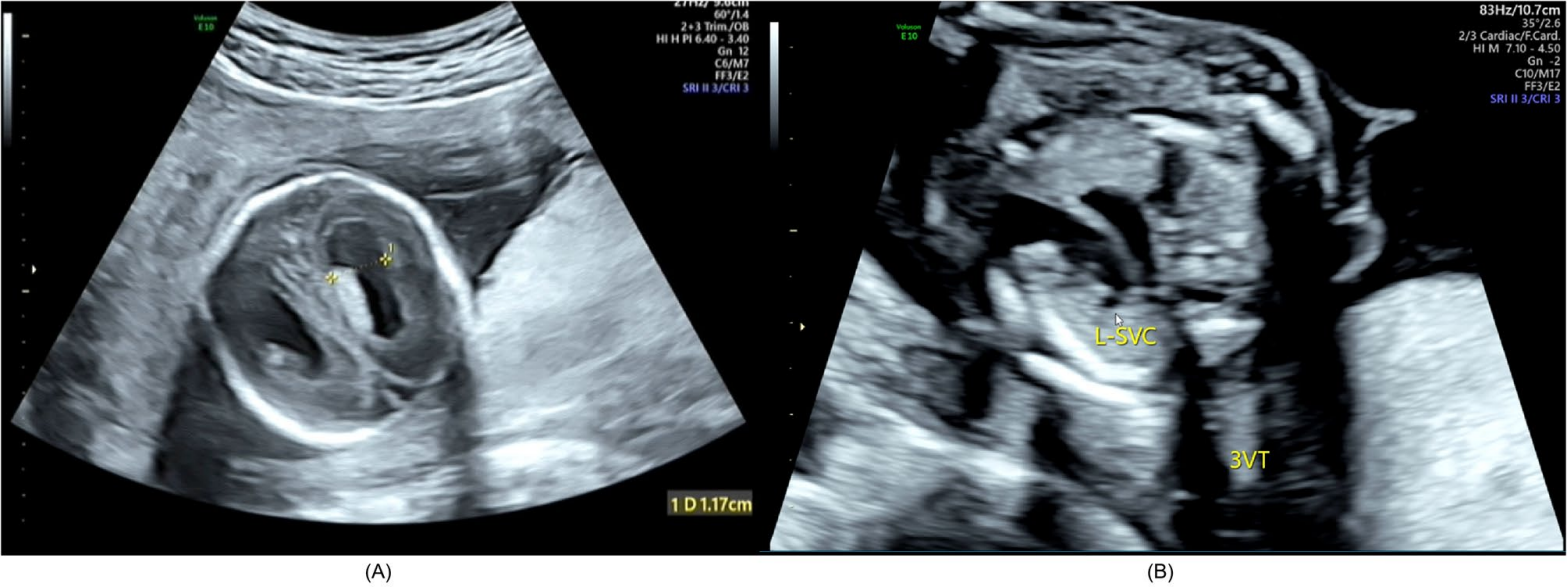
Color doppler ultrasound of II-1 during the fetal period. (**A**) Enlarged bilateral lateral ventricles; the dashed line 1 shows that the diameter of the lateral ventricle is 1.17 cm. (**B**) Persistent left superior vena cava. L-SVC: left superior vena cava, 3VT: the three vessels and trachea view

In II-1, intrauterine growth retardation was observed at 14 weeks of gestation. The enlarged bilateral lateral ventricles (Fig. 2A), hydrocephalus, and persistent left superior vena cava (Fig. 2B) were observed in II-1 at 28^+ 4^ weeks of gestation. II-1 was delivered as an extremely low birth weight infant, weighing 650 g. The Apgar scores at 1, 5, and 10 min were recorded as 4-6-6. Assessments of bodily systems were conducted within the first day of birth in order to exclude any concurrent congenital conditions. He exhibited mild tricuspid insufficiency and pulmonary arterial hypertension as along with neonatal respiratory distress and asphyxia at birth and stayed alive on a life support machine. Furthermore, PDA (4.6 mm) and right atrioventricular enlargement (cross diameter, 11 mm) were observed in II-1. At 18 days of age, the ductus arteriosus of II-1 remained patent, his condition deteriorated, and he developed hemodynamic deterioration and multiorgan failure, from which he ultimately died. Immunohistochemical examination of the lung tissue of II-1 was performed, which indicated congenital pulmonary fibrosis.

II-2’s prenatal ultrasound revealed evidence of intrauterine growth restriction. He was born with a weight of 1230 g and the Apgar scores at 1, 5, and 10 min were recorded as 6-6-8. II-2 also exhibited neonatal respiratory distress and asphyxia at birth and stayed alive on a life support machine. The color doppler echocardiography results for II-2 revealed patent ductus arteriosus (approximately 4.7 mm) with horizontal bidirectional shunt, atrial horizontal bidirectional shunt (approximately 2.8 mm), mild tricuspid insufficiency resulting in moderate pulmonary hypertension, normal range measurement of left ventricular systolic function. Functional closure of the arterial duct in II-2 was verified after administration of ibuprofen (10 mg/kg/d) for 3 days at the age of 15 days. Redundant sigmoid colon was found in II-2 during the neonatal period. Ultrasounds of the abdomen, urinary system, and craniocerebral system showed no further abnormalities. After 50 days of hospitalization, II-2 was discharged and showed no abnormality after a 12-month follow-up. All clinical manifestations of the twins are shown and compared in Table 1.

**Table 1:** Clinical phenotypes of the twins and previously reported cases with *MYH11* variants

Clinical phenotypes	II-1	II-2	Previously reported cases
Prenatal stage			
Intrauterine growth retardation	+	-	
Bilateral lateral ventricle dilation	+	-	[26]
Persistent left superior vena cava	+	-	
Neonatal stage			
Premature infant	+	+	[26, 27]
Twin-to-twin transfusion syndrome	+	+	
Neonatal respiratory distress syndrome	+	+	[26, 27]
Neonatal asphyxia	+	+	
Neonatal pneumonia	+	+	
Neonatal anemia	+	+	
Persistent pulmonary hypertension	+	+	[26]
Hyperbilirubinemia	+	+	
Patent ductus arteriosus	+	+	[12, 14, 19, 21, 26]
Hypocalcemia	+	+	
Congenital pulmonary fibrosis	+	-	
Mixed acid–base imbalance	+	+	
Neonatal septicemia	+	-	
Respiratory failure of newborn	+	-	
Cardiac failure	+	-	
Bronchopulmonary dysplasia	-	+	[26]
Redundant sigmoid colon	-	+	[26]
Other common phenotypes			
Visceral myopathy 2	-	-	[14, 15, 28]
Thoracic aortic aneurysm/dilation	-	-	[19]
Megacystis–microcolon–intestinal hypoperistalsis syndrome	-	-	[26]
Intracranial vascular malformations	-	-	[12, 21]

### A novel variant was identified in the monozygotic twins

A novel heterozygous missense variant in the *MYH11* gene [NM_002474.2: c.766 A > G p. (Ile256Val)] was identified in the proband (II-1). Sanger sequencing confirmed that the same variant was present in both II-1 and II-2, but not in the parents (Fig. 1). The variant was classified as a likely pathogenic variation according to ACMG/AMP guidelines [16]. The variant was considered to be deleterious based on the following evidence: [1] this variant was not found in the parents and occurred *de novo* (PS2); [2] negative results were obtained in searches of the 1000 Genomes Project, Genome Aggregation Database, Exome Sequencing Project, and Exome Aggregation Consortium (PM2); and [3] multiple *in silico* tools, for example the CADD phred-like score is 21.9, indicated a deleterious effect (PP3).

### Phenotypes caused by pathogenic variants in *MYH11* may be associated with the variant type

**Fig. 3:**
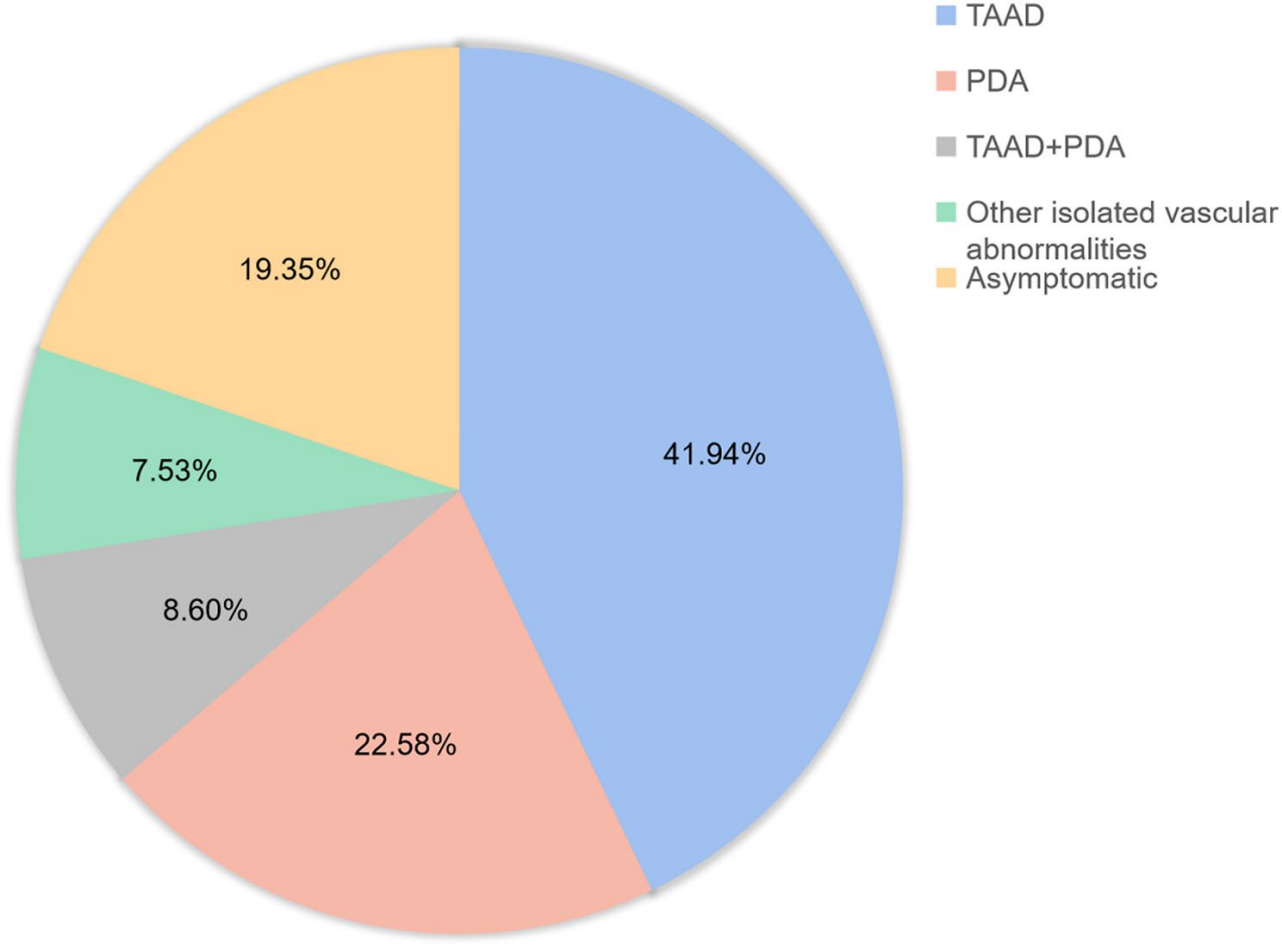
The distribution of phenotypes of the hereditary TAAD/PDA family with *MYH11* variants

**Fig. 4:**
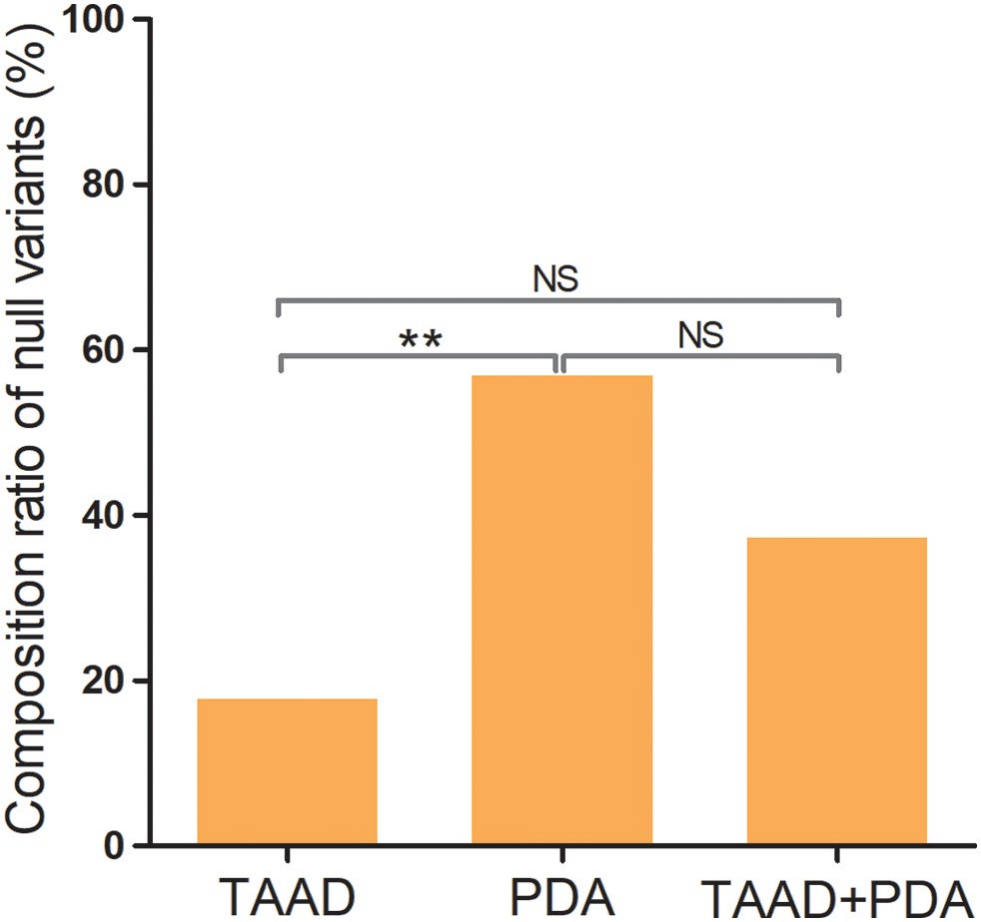
The component ratios of null variants in *MYH11* among groups of TAAD, PDA, and TAAD + PDA. The component ratios of null variants were significantly different between groups TAAD and PDA (*P* = 0.006), but there were no differences between the TAAD and TAAD + PDA groups, or between PDA and TAAD + PDA groups

**Fig. 5:**
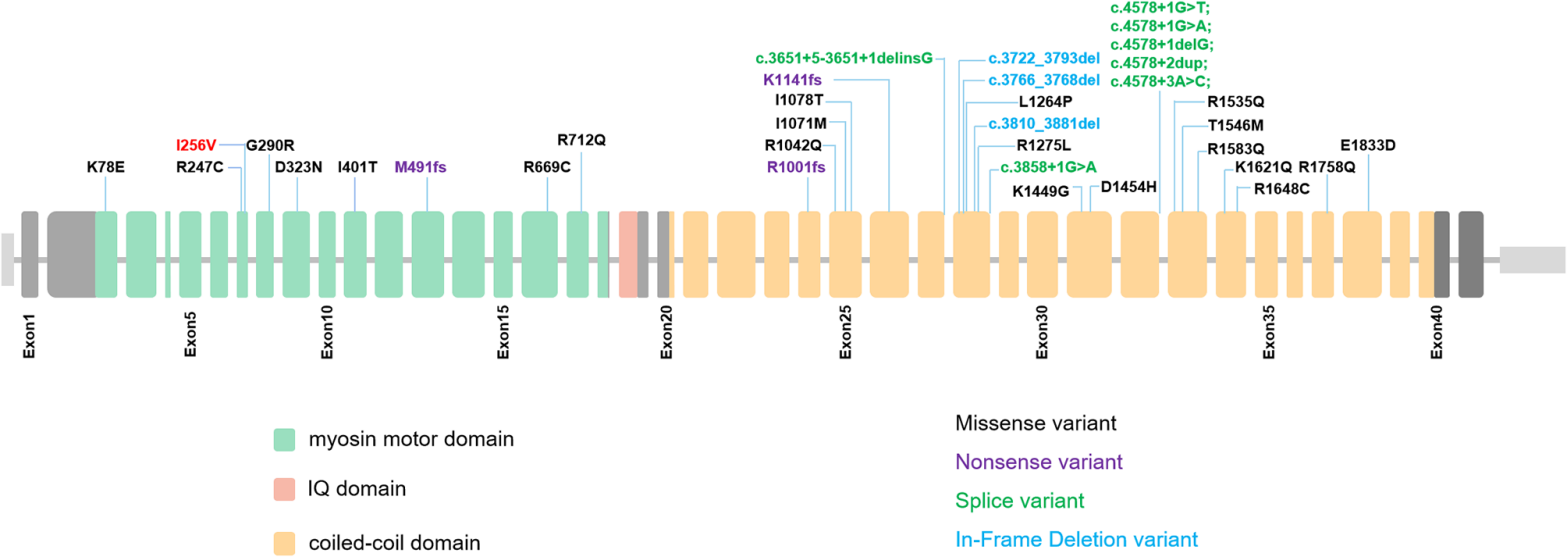
Schematic representation of the protein structure of *MYH11* (NM_002474.2). The distribution of different types of pathogenic variants in *MYH11* was shown. The variant (I256V) was identified in our monozygotic twins

The penetrance of *MYH11* is 82.35% (84/102) in the reported cases (*n* = 102). The most common manifestation caused by *MYH11* variants was TAAD (41.18%, 42/102), followed by PDA (22.55%, 23/102), compound TAAD and PDA (TAAD + PDA) (9.80%, 10/102), and other vascular abnormalities (8.82%, 9/102) (Fig. 3). In the case with different phenotypes, 35 variants have been identified, including null variants (nonsense and splicing site, *n* = 10), missense variants (*n* = 22) and in-frame deletion variants (*n* = 3, which was excluded in futher analyses). However, the analysis of the groups with TAAD, PDA, and TAAD + PDA showed that the constituent ratios of null or missense variants were significantly different (*P* = 0.01). Further pairwise comparison among these three groups with various phenotypes showed that the constituent ratios of null variants were significantly different between groups of TAAD (8.60%) and PDA (43.8%, *P* = 0.006), but there were no differences between the TAAD and TAAD + PDA (28.6%) groups (*P* = 0.188), or PDA and TAAD + PDA groups (*P* = 0.657) (Fig. 4). These identified variants of *MYH11* were distributed separately in the coiled-coil domain (74.3%, 26/35) and myosin motor domain (25.7%, 9/35) (Fig. 5). However, the constituent ratios of the variant located in different domains of *MYH11* were not significantly different among the groups with TAAD, PDA, and TAAD + PDA (*P* = 0.778). Moreover, we noticed that individuals with some specific variants show highly variable clinical features even asymptomatic, indicating interfamilial or intrafamilial variable expressivity.

## Discussion

TAAD/PDA caused by variants in genes that encode the contractile proteins of vascular SMCs presents variable expressivity and penetrance. *MYH11* encodes a contractile protein in arterial vascular SMCs which plays an important role in maintaining the stability of the vascular wall. However, the genotype–phenotype correlation of *MYH11* is still unclear. We describe a pair of monozygotic twins with highly variable clinical features and outcomes. Both of the monozygotic twins presented with PDA, pulmonary hypoplasia, and pulmonary hypertension. The proband developed heart and brain abnormalities during the fetal stage and died in the neonatal period, whereas his twin sibling was cured and continued to grow normally. The same novel heterozygous missense variant in *MYH11* was identified in the monozygotic twins. Based on the phenotypic and genetic analysis of the monozygotic twins, further genotype-phenotype association analysis of *MYH11* was performed by comparing previously reported cases. The analysis of the genotype-phenotype correlation of *MYH11* indicated that the phenotypes caused by pathogenic variants in *MYH11* may be related to different types of variants. The missense variants in *MYH11* were more frequent in the group with TAAD, whereas the null variants in *MYH11* were more frequent in the group with PDA.

*MYH11* was the first gene defect reported in the gene family of smooth muscle contraction-associated vasculopathy. The pathogenic variant in *MYH11* usually contributes to TAAD/PDA with variable expressivity and penetrance. Marked aortic stiffness was found in individuals (including asymptomatic) with the heterozygous variant, indicating that haploinsufficiency may be the mechanism of the variants in *MYH11* [9, 17]. The interaction of wild-type and mutant proteins in co-immunoprecipitation testing suggested that the mechanism of the variants in *MYH11* can also be explained by dominant negative effect [9]. Compound heterozygotic variants (L1264P and R1275L) on the same allele of *MYH11* were identified in a TAAD/PDA family, wherein two members displayed TAAD + PDA, whereas the other two members displayed isolated TAAD [15]. The L1264P variant was also identified in another family, wherein five individuals bearing this variant presented with isolated TAAD [18]. Several possible mechanisms, including potential genetic modifiers co-operate with the variant to regulate the expression of *MYH11*, were proposed to explain that the same L1264P variant resulted in different phenotypes [15]. Moreover, Harakalova M et al. [19] had reported two TAAD/PDA families with various phenotypes, presenting incomplete co-segregation of TAAD/PDA with variants in *MYH11*. The mechanism of such phenomenon could be explained by the oligogenic model [19]. Markedly decreased cell apoptosis and increased proliferation were found in the aortic SMCs of Myh11^−/−^ mice [20], and the case with arterial stenosis in the intracranial vessel involvement caused by variants of *MYH11* has been reported recently [21], indicating that there are other potential mechanisms of variants in *MYH11* which remain to be explored. The mechanism by which variants in *MYH11* result in TAAD/PDA is complex, and the genotype–phenotype correlation of *MYH11* has not been clearly determined.

The analysis of the genotype-phenotype correlation of *MYH11* indicated that phenotypes caused by pathogenic variants in *MYH11* may be associated with the type of variant. Missense variations of *MYH11* are highly likely to be associated with TAAD, whereas PDA is mostly caused by null variations. In general, TAAD is found in older adults, whereas PDA occurs immediately after birth. According to the results of our analysis of genotype–phenotype correlation, we speculate that the distinct phenotypes caused by variants in *MYH11* are due to various effects from different types of variants. Since the missense variant has a relatively minor negative effect on *MYH11*, it mainly causes TAAD, which requires a certain amount of time to accumulate before disease onset. In contrast, the null variant, which strongly disrupts the function of *MYH11*, results in PDA, an early-onset disease. However, TAAD associated with variants in *MYH11* probably present variable disease onset and progression; thus, there may have been an insufficient amount of time for noticeable defects to occur in our patients. The risk of occurrence of TAAD suggests that II-2 should undergo regular cardiovascular surveillance in the future. Rare pathogenic variants mainly affect the myosin head and coiled-coil domain of *MYH11*.

Phenotypes caused by different variants located in different domains of *MYH11* were not significantly different. Whether the domains of *MYH11* are completely unrelated or potentially associated remains to be elucidated. Further research is necessary to reveal the correlation between domains of *MYH11* and TAAD/PDA. We cannot exclude the influence of confounding factors, such as the age of patients in the analysis of genotype–phenotype correlations. Moreover, bias could not be avoided in the statistical analysis owing to the limited number of cases collected and the high pedigree weight among the patients. However, we found in more than one case, that the same variant caused a scattered distribution of phenotypes in the same family [18]. A genetic predisposition to PDA has been demonstrated by some studies with animal and human models [22, 23]. The monozygotic twins also remind us that genetic factors should not be overlooked due to the possibility of a genetic link to PDA in premature infants.

The interfamilial or intrafamilial variable expressivity of *MYH11* has been primarily studied. Several likely factors, including allelic variation, oligogenic models, environmental factors, modifier genes, and complex genetic and environmental interaction variability have been proposed to explain phenotypic variability of *MYH11* [9, 18, 19]. Surprisingly, despite the similar environmental effects and genetic backgrounds in the monozygotic twins, different phenotypes and variable expressivity were observed. It indicates that there are other potential modifiers and complex mechanisms beside the genetic variants that influence the expression of *MYH11*. A previous study showed that the random monoallelic expression of dosage-sensitive genes may play a crucial role in phenotypic variability [24]. Random monoallelic expression of *MYH11* may explain the complex phenotypic variability in our patients. Mosaic expression may have emerged, owing to the expression of random and dynamic monoallelic genes. The expression ratios of wild-type and mutant alleles differ in the cells of different tissues or organs. Moreover, different spatio-temporal expression patterns may confer diverse function for specific genes [25]; specific expression of *MYH11* in different organs or tissues at different developmental stages may also be the cause of highly variable clinical features and outcomes in our monozygotic twins.

In conclusion, this study expands the mutational and phenotype spectra of *MYH11*, thereby speculating that the phenotypes caused by pathogenic variants in *MYH11* may be associated with variant type. Moreover, we believe that different random monoallelic expression and spatio-temporal expression are the possible reasons for the complex penetrance and variable expressivity of *MYH11*. However, the genotype–phenotype correlations of *MYH11* could not be completely established, owing to the limited number of reported cases. Further investigation is required to reveal the complex genotype–phenotype correlation of *MYH11*.

### Electronic supplementary material

Below is the link to the electronic supplementary material.

Supplementary Material 1

Supplementary Material 2

## Data Availability

The datasets that support the findings of this study can be requested from the corresponding author. The data are also publicly available from the ClinVar database (submission id: SUB12969714).
