# Supplementary material for: Complex genotype–phenotype correlation of MYH11: new insights from monozygotic twins with highly variable expressivity and outcomes
Source: BMC Med Genomics. 2024 May 21;17:135. doi: 10.1186/s12920-024-01908-5 (PMC11110423; doi:10.1186/s12920-024-01908-5)
Supplement: Supplementary file 1 — Supplementary Material 1 [file 12920_2024_1908_MOESM1_ESM.pdf]

Supplementary Material 1

Figure 1. Short tandem repeat (STR) sequencing of the family including non-consanguineous parents and monozygotic twins. (A) Four STR loci including D18S1002, D18S391, D18S535 and D18S386 was identified in I-1, I-2, II-1 and II-2. The result showed that II-1 and II-2 have the same allele, and this pair of alleles comes from I-1 and I-2 respectively, which indicated that II-1 and II-2 are monozygotic twins, and confirms their genetic relationship with I-1 and I-2. (B) Four STR loci including D13S628, D13S742, D13S634 and D13S305 was identified in I-1, I-2, II-1 and II-2. The result showed that II-1 and II-2 have the same allele, and this pair of alleles comes from I-1 and I-2 respectively, which indicated that II-1 and II-2 are monozygotic twins, and confirms their genetic relationship with I-1 and I-2.

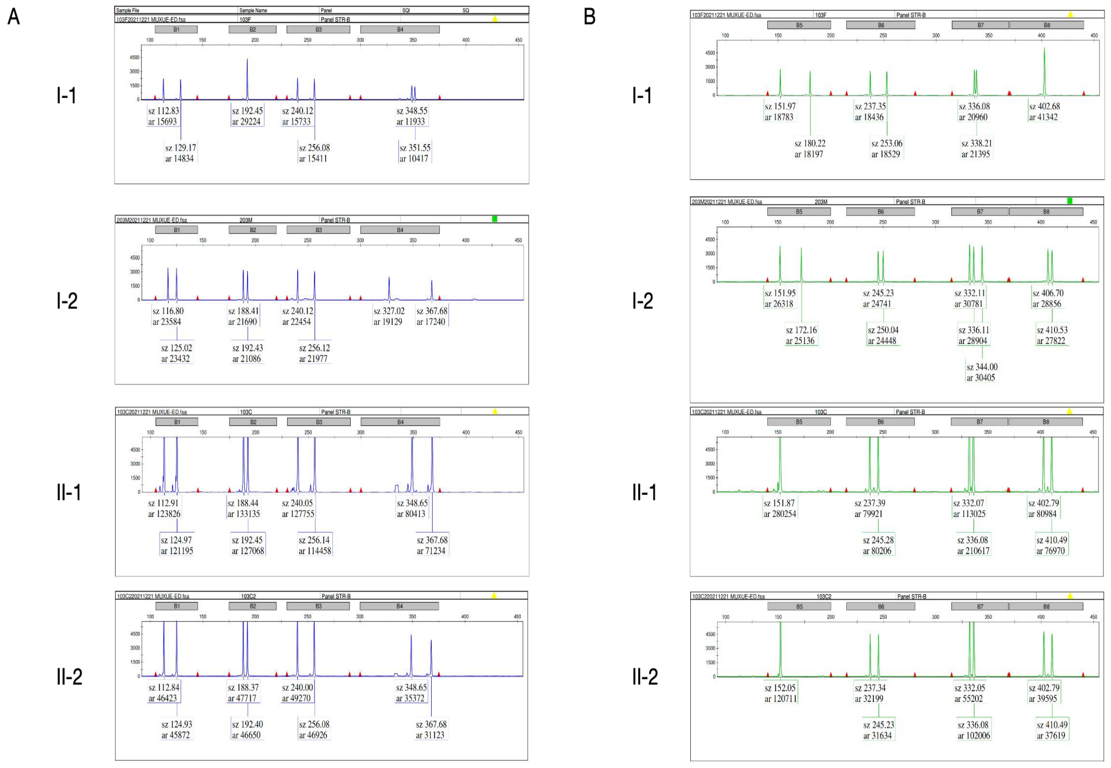

Figure 1: Short tandem repeat (STR) sequencing of the family including non-consanguineous parents(I-1 and I-2) and monozygotic twins(II-1 and II-2) .
